# Molecular Identification of a Malaria Merozoite Surface Sheddase

**DOI:** 10.1371/journal.ppat.0010029

**Published:** 2005-11-25

**Authors:** Philippa K Harris, Sharon Yeoh, Anton R Dluzewski, Rebecca A O'Donnell, Chrislaine Withers-Martinez, Fiona Hackett, Lawrence H Bannister, Graham H Mitchell, Michael J Blackman

**Affiliations:** 1 Division of Parasitology, National Institute for Medical Research, London, United Kingdom; 2 Department of Immunobiology, Guy's, King's and St. Thomas' Hospitals School of Medicine, London, United Kingdom; 3 Wolfson Centre, Guy's, King's and St. Thomas' Hospitals School of Biomedical and Life Sciences, London, United Kingdom; Stanford University, United States of America

## Abstract

Proteolytic shedding of surface proteins during invasion by apicomplexan parasites is a widespread phenomenon, thought to represent a mechanism by which the parasites disengage adhesin-receptor complexes in order to gain entry into their host cell. Erythrocyte invasion by merozoites of the malaria parasite *Plasmodium falciparum* requires the shedding of ectodomain components of two essential surface proteins, called MSP1 and AMA1. Both are released by the same merozoite surface “sheddase,” but the molecular identity and mode of action of this protease is unknown. Here we identify it as PfSUB2, an integral membrane subtilisin-like protease (subtilase). We show that PfSUB2 is stored in apical secretory organelles called micronemes. Upon merozoite release it is secreted onto the parasite surface and translocates to its posterior pole in an actin-dependent manner, a trafficking pattern predicted of the sheddase. Subtilase propeptides are usually selective inhibitors of their cognate protease, and the PfSUB2 propeptide is no exception; we show that recombinant PfSUB2 propeptide binds specifically to mature parasite-derived PfSUB2 and is a potent, selective inhibitor of MSP1 and AMA1 shedding, directly establishing PfSUB2 as the sheddase. PfSUB2 is a new potential target for drugs designed to prevent erythrocyte invasion by the malaria parasite.

## Synopsis

Malaria causes immense suffering and loss of life across the globe. In the face of growing resistance to available drugs and no licensed vaccine, new approaches are urgently required to tackle its control. Fundamental to these is an improved understanding of the basic biology of the malaria parasite. The parasite invades and replicates within red blood cells. During invasion a number of important proteins need to be shed from the parasite surface, probably in order to disengage the adhesive interactions that enable initial binding. Shedding of these surface proteins is achieved by a parasite enzyme called a protease, and compounds or antibodies that block the action of this protease prevent invasion, killing the parasite. Here the authors identify this protease as PfSUB2, a large, membrane-bound member of the subtilisin-like protease superfamily. They find that PfSUB2 is secreted from apical organelles called micronemes at the point of invasion to migrate rearwards over the surface of the parasite, and that a protein designed to be a specific inhibitor of PfSUB2 potently prevents shedding of parasite surface proteins. This work sets the scene for the development of inhibitors of PfSUB2 as a new generation of antimalarial drugs.

## Introduction

Malaria is a devastating global health problem, responsible for up to 3 million deaths annually [1]. The disease results from cyclical replication within erythrocytes of protozoan parasites of the genus *Plasmodium*. The parasite divides asexually within its host cell to produce a number of progeny merozoites. Upon eventual rupture of the schizont, these are released to rapidly invade fresh red cells and perpetuate the cycle. Like all apicomplexan parasites, the *Plasmodium* merozoite enters its host cell by an active invasion process that is mediated by adhesive receptor–ligand interactions and driven by an actinomyosin motor [2]. Light and electron microscopic studies have shown that initial attachment to the host erythrocyte is followed by reorientation of the merozoite such that its apical end contacts the cell surface. This results in the formation of an irreversible zone of contact, or tight junction, between the apical prominence and the host cell surface. The host cell membrane then invaginates, forming a parasitophorous vacuole (PV) into which the parasite is propelled; in the process, the junction sweeps around the periphery of the parasite with concomitant “shaving” of bristle-like structures from the parasite surface [3,4], eventually sealing behind the intracellular parasite. The initial low-affinity binding appears to be mediated by a large, glycosylphosphatidyl inositol (GPI)-anchored protein complex which is uniformly distributed around the parasite surface and is composed of fragments of merozoite surface protein-1 (MSP1) plus associated partner proteins [5–7]. Many subsequent interactions in the invasion pathway are mediated by proteins released from micronemes, secretory vesicles at the apical end of the merozoite [8]. One of these proteins, apical membrane antigen-1 (AMA1), is a type I integral membrane protein that is secreted onto the merozoite surface just prior to interaction with the host cell and may play a role in reorientation, junction formation, or government of the release of a second set of apical organelles called rhoptries [9–11]. Both MSP1 and AMA1 play essential roles in the blood-stage cycle of the malaria parasite [12,13].

During invasion both AMA1 and the MSP1 complex are quantitatively shed from the parasite surface, in each case as a result of a single proteolytic cleavage at a juxtamembrane site. Shedding of MSP1 results from cleavage just distal to a tandem EGF (epidermal growth factor)-like domain called MSP1_19_ at its C-terminus [14]. MSP1_19_ remains bound to the parasite surface via its GPI anchor and is the only part of the MSP1 complex to be carried into the host cell. AMA1 is cleaved precisely 29 residues away from the transmembrane domain (TMD), releasing the bulk of the ectodomain and resulting in just the juxtamembrane “stub” being carried into the host cell with its cognate TMD and cytoplasmic domain [15–17]. Shedding of these proteins is required for productive invasion [7,18,19], and may be important to release adhesive interactions between the parasite and host cell surface in order to allow unimpeded passage into the nascent PV [20]. The MSP1 complex is an abundant merozoite component, and together with AMA1 likely corresponds to the surface structures shed adjacent to the moving junction. Importantly, shedding of both AMA1 and the MSP1 complex can occur even in the absence of invasion, and a simple assay based on the use of isolated merozoites has shown that both proteins are shed by the same parasite-derived, membrane-bound, calcium-dependent serine protease, called merozoite surface sheddase, or MESH [16,20]. MESH is active against its physiological substrates only when in the same membrane [21]. Its molecular identification has proved elusive, but the accumulated microscopic and biochemical evidence suggests that at invasion it must distribute across the surface of the parasite, probably localising at the moving junction [7,20]. We and others have previously identified a large, membrane-bound subtilisin-like serine protease called SUB2 that is expressed in the late stages of intraerythrocytic development and accumulates within the apical domain of the merozoite [22,23]. The enzyme has not been expressed in a recombinant, enzymatically active form, but SUB2 is conserved throughout *Plasmodium,* and attempts to disrupt both the *Plasmodium falciparum* gene *(pfsub2)* and that of the rodent malaria *P. berghei* have been unsuccessful [24,25], indicating an essential function within the blood-stage cycle. Here we present the first experimental evidence that PfSUB2 is MESH, and show that it has the remarkable capacity to translocate across the merozoite surface exactly as predicted.

## Results

### Epitope Tagging of PfSUB2 by Targeted Homologous Recombination

**Figure 1:**
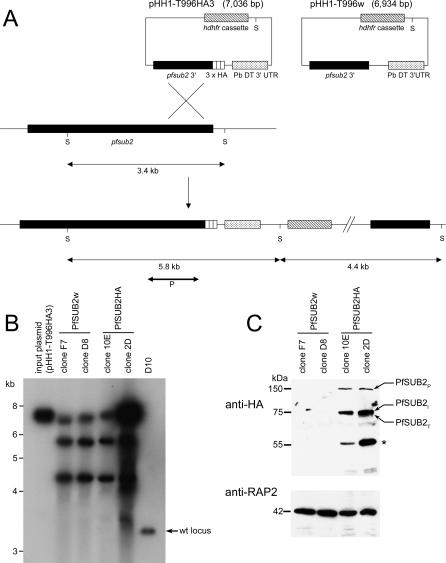
Epitope Tagging of PfSUB2 (A) Schematic depiction of integration plasmids pHH1-T996HA3 and pHH1-T996w, and single-crossover homologous recombination events (for clarity shown only for pHH1-T996HA3). Integration reconstitutes a full-length *pfsub2* gene either with or without a triple HA tag at the 3′ end. Correct transcription termination and polyadenylation of this gene is regulated by the presence of the *P. berghei* dihydrofolate reductase 3′ UTR (PbDT 3′ UTR). *hdhfr,* human dihydrofolate reductase, conferring resistance to the antifolate WR99210. S, indicates the ScaI sites. P indicates the position and size of the probe used for Southern analysis. (B) Southern blots of ScaI-digested input plasmid pHH1-T996HA3 or genomic DNA from PfSUB2w clones F7 and D8, PfSUB2HA clones 10E and 2D, and wild-type D10 parasites. The position of the genomic 3.4-kb fragment derived from the wild-type (wt) *pfsub2* locus is arrowed. Its disappearance from all the transgenic clones indicates disruption of this locus, whilst the appearance of the additional species at 5.8 kb and 4.4 kb indicates integration as predicted. The fragments seen at the position of full-length input plasmid derive from integration of more than one copy of the entire plasmid, as described in the text. (C) Western blot of transgenic clones using anti-HA mAb 3F10 (identical results were obtained using anti-HA mAb 12CA5; not shown) or anti-RAP2 mAb H5, used as a loading control. Identities of the authentic PfSUB2 species are indicated, and the 55-kDa PfSUB2 degradation product (the abundance of which varied widely between experiments, being most prominent in non-ionic detergent extracts incubated for prolonged periods) is marked with an asterisk. Note that the predicted molecular mass of an HA-tagged minimal PfSUB2 catalytic domain (extending from the catalytic Asp755 to the end of the C-terminal tag) is approximately 70 kDa. Molecular masses are in kDa.

PfSUB2 is a poorly abundant parasite protein, hindering our earlier attempts at precise sub-cellular localization of the mature protein. To overcome this obstacle, we set out to modify the endogenous *pfsub2* gene by fusing it to a sequence encoding three consecutive haemagglutinin (HA) epitope tags using targeted homologous recombination. Parasites transfected with construct pHH1-T996HA3 and the control plasmid pHH1-T996w (Figure 1A) were cultured in the presence of the antifolate WR99210 followed by rounds of drug cycling to remove parasites harbouring free episomes (which are rapidly lost in the absence of drug pressure). The resulting parasite lines, called PfSUB2HA and PfSUB2w, were cloned by limiting dilution and maintained in the presence of WR99210. Southern blot analysis (Figure 1B) showed that all transgenic clones contained more than one copy of the targeting plasmid integrated into the *pfsub2* locus, as is common in single-crossover homologous recombination in *P. falciparum* as a result of the plasmids being maintained as concatamers (e.g., [13]). Note that despite this, only one copy of the modified gene is expected to be functional, because all downstream copies contain either the targeting fragment only or a promoterless, truncated remnant of the endogenous locus lacking the epitope tag. In over 6 mo of continuous culture, none of the clones exhibited any growth defect compared to the parental D10 clone (not shown), indicating that neither the addition of the HA tags to the C-terminus of PfSUB2 nor replacement of the *pfsub2* 3′ UTR with the heterologous *P. berghei* sequence was detrimental to parasite replication.

Analysis of the transgenic clones by Western blot using two different HA-specific monoclonal antibodies (mAbs), 12CA5 and 3F10, confirmed the in-frame fusion of the epitope tag to PfSUB2. Pulse-chase studies have shown that PfSUB2 maturation involves two post-translational processing steps in which the approximately 150-kDa primary translation product (called PfSUB2_P_) is converted first to a 75-kDa intermediate form (PfSUB2_I_) and finally to a 72-kDa terminal intracellular form (PfSUB2_T_) [23,25]. All were detected in schizont extracts probed with the anti-HA mAbs (Figure 1C; the closely spaced PfSUB2_I_/PfSUB2_T_ doublet is not easily discernible here, but see below), consistent with our earlier evidence that maturation of PfSUB2 involves truncation from the N-terminus of the protein. An additional anti-HA reactive species of 55 kDa, also often evident on Western blots, has previously been observed as a minor component in pulse-chase experiments, and is likely a non-specific degradation product of PfSUB2.

### PfSUB2 Is a Microneme Protein

**Figure 2:**
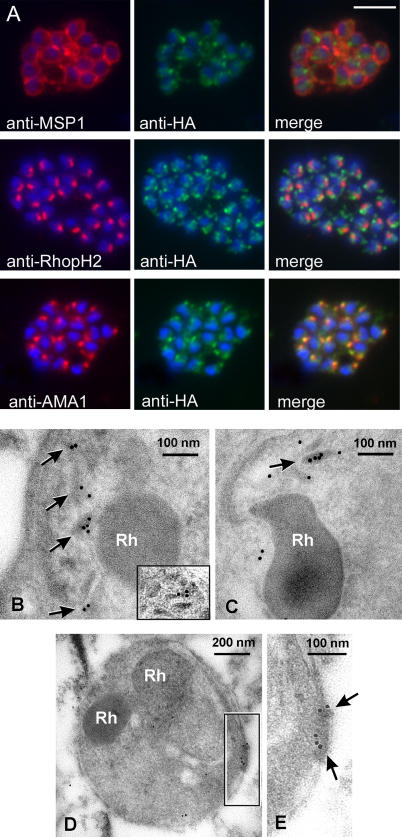
PfSUB2 Is a Microneme Protein (A) IFA images of schizonts of PfSUB2HA clone 2D dual-labelled with mAbs X509 (anti-MSP1), 61.3 (anti-RhopH2), or 4G2 (anti-AMA1), plus in each case mAb 3F10 (anti-HA) The anti-HA signal co-localised only with the anti-AMA1 signal. Identical results were obtained with the uncloned transgenic PfSUB2HA line, and/or when anti-RAP2 mAb H5 was used as the rhoptry marker instead of mAb 61.3 (not shown). Parasite nuclei are stained throughout with DAPI (blue). Scale bar represents 2 μm. (B and C) Electron micrographs showing immuno-gold labelling of micronemes within late-stage schizonts of PfSUB2HA clone 2D using: (B) anti-HA mAb 3F10, detecting epitope-tagged PfSUB2; and (C) a polyclonal antibody specific for PfAMA1. The inset in (B) shows another example of micronemal staining with mAb 3F10 from another schizont. Rh, rhoptry. (D and E) Posterior labelling of with anti-HA mAb 3F10 in a free merozoite of PfSUB2HA clone 2D. Arrows indicate immuno-gold labelling. Rh, rhoptry.

To establish the sub-cellular location of PfSUB2, parasites were then analysed by indirect immunofluorescence (IFA) using both of the anti-HA mAbs. Neither mAb showed any reactivity with any stages of the PfSUB2w line or clones thereof, or with the parental D10 clone (not shown). In contrast, both mAbs produced a strong punctate pattern in mature schizonts of both the uncloned PfSUB2HA line (not shown) and its clones. To examine this localisation in detail, dual labelling was performed using mAbs specific for a plasma membrane marker (MSP1), two different rhoptry markers (RhopH2 and RAP2; data not shown for RAP2), and a microneme protein (AMA1). The results were clear (Figure 2A); the anti-HA signal was quite different from that of MSP1, it was adjacent to but distinct from that of the rhoptry-specific signals, but it co-localised completely with that of AMA1, indicating that in mature schizonts, PfSUB2 accumulates in micronemes.

These findings were confirmed by immuno-electron microscopy (IEM) using the anti-HA mAb 3F10, which showed light but consistent labelling of typical elongated micronemes within late-stage schizonts (Figure 2B), similar to that of AMA1 (Figure 2C). No labelling with mAb 3F10 was observed in less-mature schizont stages lacking micronemes, or apically in free merozoites, and labelling was also absent from wild-type schizont controls (not shown). There was no evidence of any dense granule labelling with the anti-HA antibody.

### PfSUB2 Is Secreted to Translocate across the Surface of Free Merozoites

**Figure 3:**
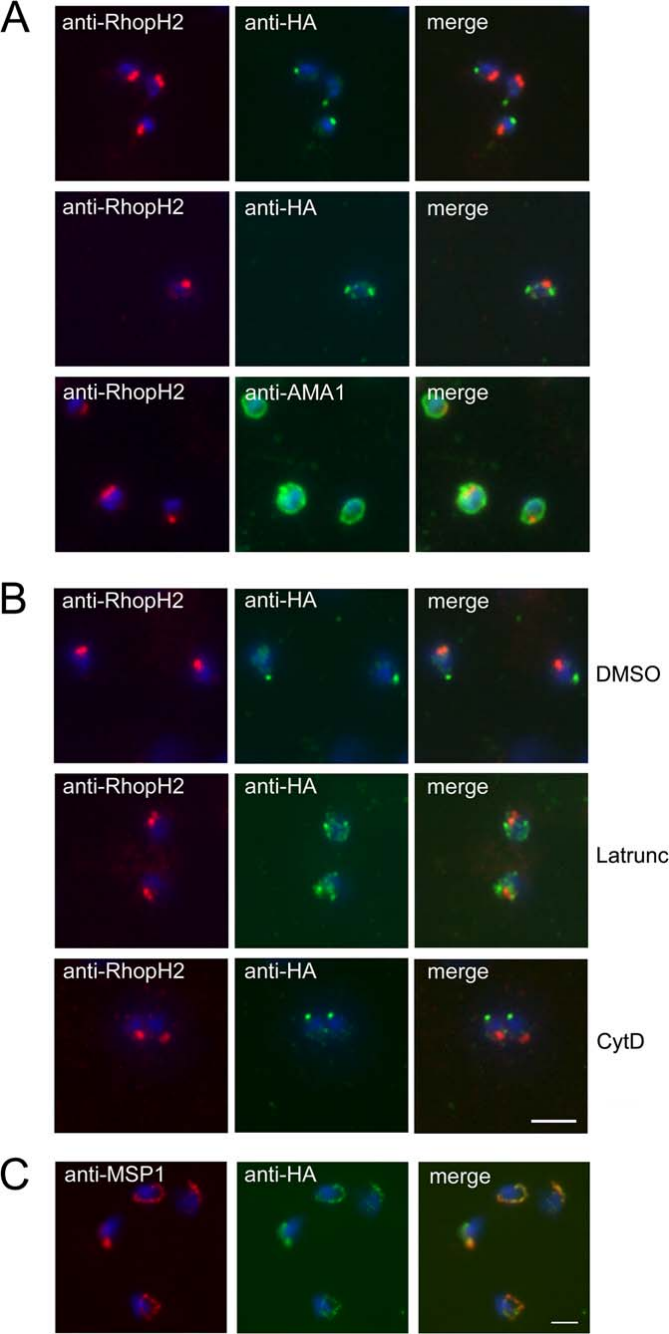
PfSUB2 Redistributes to the Free Merozoite Posterior in an Actin-Dependent Manner (A) IFA images of free merozoites of PfSUB2HA clone 2D dual-labelled with mAb 61.3 (anti-RhopH2) and either mAb 3F10 (anti-HA) or 4G2 (anti-AMA1). The anti-HA signal formed either a single punctate signal at the extreme posterior of the merozoite (top), or twin foci lateral to its anterior–posterior axis (middle). Identical results were obtained using PfSUB2HA clone 10E, or using anti-RAP2 mAb H5 as the rhoptry marker (not shown). (B) Latrunculin inhibits rearward translocation of PfSUB2. Merozoites of PfSUB2HA clone 2D released into medium containing 1% (v/v) DMSO only (solvent control), or 5 μM latrunculin A (Latrunc), or 4 μM cytochalasin D (Cyt D), probed as above with mAb 61.3 (anti-RhopH2) and mAb 3F10 (anti-HA). Note that neither compound had any effect on the efficiency of schizont rupture and merozoite release, nor on AMA1 relocalisation, but both blocked erythrocyte invasion by more than 95% at the concentrations used (not shown). (C) PfSUB2 remains at the plasma membrane of the newly invaded parasite. Ring stages of PfSUB2HA clone 2D (≤2 h post-invasion) probed with anti-MSP1 mAb 1E1 and anti-HA mAb 3F10. Parasite nuclei are stained throughout with DAPI (blue). Scale bar represents 2 μm.

To follow the fate of PfSUB2 upon merozoite release, naturally released merozoites were next examined by IFA as described above. As with intracellular parasites in mature schizonts, all merozoites exhibited an intense, punctate anti-HA signal. Unexpectedly, however, in the great majority of cases (≥70% of free merozoites), it comprised a single tight focus of fluorescence that clearly localised to the opposite pole of the merozoite to that recognised by the anti-rhoptry mAbs. This posterior localisation was also found in the free merozoite by IEM, labelling being present just interior to the cell surface, consistent with antibodies binding to the epitope tag at the extreme C-terminus of the PfSUB2 cytoplasmic domain (Figure 2D and 2E; Figure 3A, top row). There are no known organelles situated at the posterior of the merozoite, so this suggested that, just after schizont rupture, PfSUB2 is secreted from the micronemes onto the merozoite surface and redistributed rearwards to accumulate at its posterior end. In a portion of cases (~10%), the anti-HA IFA signal presented as two separate foci that lay lateral to the anterior–posterior axis of the merozoite, suggesting the presence of a mid-bodied ring of fluorescence encircling the parasite at right angles to this axis and perhaps representing PfSUB2 en route to the posterior (Figure 3A, middle row). Only in less than 20% of cases did the anti-HA IFA signal remain adjacent to the rhoptries as reproducibly observed in intact schizonts. In contrast, the anti-AMA1 IFA signal in free merozoites took the form of a uniform circumferential pattern (Figure 3A, bottom row) as observed previously (e.g., [16]). In no case did the anti-AMA1 IFA signal concentrate at the posterior pole of the parasite as seen for the anti-HA signal. Several apicomplexan microneme proteins translocate onto the parasite surface upon release from the host cell. The best-studied example, the *Toxoplasma* adhesin TgMIC2/M2AP, is secreted spontaneously at low basal levels and capped to the posterior pole of the free tachyzoite. As the parasite invades a host cell, secretion is markedly up-regulated and driven rapidly to completion [26]. Our observations suggest that PfSUB2 behaves in a somewhat similar manner, although complete translocation of PfSUB2 can apparently occur even in the absence of host cell invasion. These findings document the first demonstration of capping of a *Plasmodium* merozoite surface protein. Importantly, the trafficking pattern observed indicates that PfSUB2 has the capacity to track along the merozoite surface, a characteristic predicted of a membrane-bound protease involved in shedding of merozoite surface proteins.

### Translocation of PfSUB2 Is Actin Dependent

The unique form of substrate-dependent gliding motility exhibited by many Apicomplexa is mediated by interactions between the cytoplasmic domains of transmembrane adhesins, such as TgMIC2/M2AP or its *Plasmodium* sporozoite homologue, TRAP, and a subplasmalemmal actinomyosin motor [2]. Compounds that interfere with this motor block motility and invasion, and prevent capping of several micronemal adhesins, though not their secretion per se (e.g., [27,28]). To test the possibility that this or a similar actin-dependent system might drive PfSUB2 translocation, we investigated the effects of latrunculin A and cytochalasin D, two inhibitors of actin polymerisation with distinct mechanisms of action. As shown in Figure 3B, 5 μM latrunculin A clearly inhibited posterior accumulation of PfSUB2 on released merozoites, with approximately 90% of free parasites exhibiting instead a diffuse distribution of the anti-HA signal at or around the apical region of the merozoite. This result indicates that rearward translocation of PfSUB2 is actin dependent. In contrast, cytochalasin D had no effect on PfSUB2 capping, even at concentrations as high as 4 μM (Figure 3B). This was surprising given the known inhibitory effect of cytochalasins on invasion by the malaria merozoite [29], but identical results were obtained in three independent experiments using two different batches of the drug.

### PfSUB2 Remains on the Plasma Membrane of Invading Parasites

Probing newly invaded ring-stage parasites from highly synchronised cultures of the PfSUB2HA clones with mAb 3F10 produced a “ring-shaped” signal that co-localised with that of a mAb specific for MSP1_19_, the fragment of MSP1 that remains on the parasite surface after invasion (Figure 3C). By 4–6 h post invasion, the anti-HA IFA signal was no longer detectable, only to appear again in a punctate form in the late stages of schizont maturation (not shown). PfSUB2 expression peaks in the latter stages of intraerythrocytic maturation [22,30,31], so the signal observed in early rings must derive from PfSUB2 carried into the cell on the merozoite surface. Collectively, the developmental profile of PfSUB2 expression and sub-cellular trafficking, its ability to mobilise onto and across the merozoite surface, and its presence at the plasma membrane of newly invaded ring stages are entirely consistent with an involvement in MESH activity.

### Recombinant PfSUB2 Propeptide Is Folded and Binds Specifically to Mature Parasite-Derived PfSUB2

Subtilases are synthesised as zymogens that comprise minimally a secretory signal peptide, a propeptide, and a catalytic domain. The propeptide acts as an intramolecular chaperone, being essential for folding of the catalytic domain. Subtilase propeptides are also highly potent, selective inhibitors of their cognate proteases (e.g., [32–34]), a property that is probably important in regulating subtilase activation during secretion. Prompted by the finding that PfSUB2 is actively distributed across the free merozoite surface, we investigated whether its propeptide could interfere with MESH activity.

**Figure 4:**
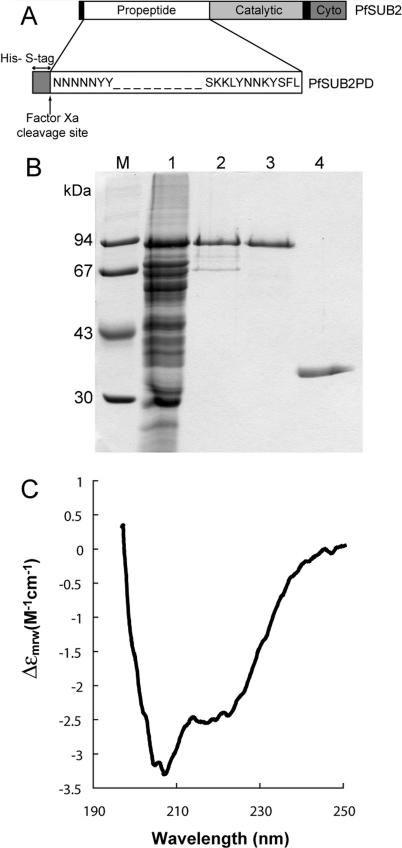
Recombinant PfSUB2 Propeptide Is Soluble and Structured (A) Schematic of the PfSUB2PD construct. Full-length PfSUB2 is represented with the secretory signal peptide and TMD indicated in black and the catalytic and cytoplasmic domains in shades of grey. PfSUB2PD possesses a cleavable 46-residue N-terminal fusion peptide containing a His6 and an S-tag, shown in dark grey. (B) Coomassie-stained SDS PAGE gel showing stages in the purification of PfSUB2PD. Lane 1, Ni-NTA agarose eluate enriched in PfSUB2PD; lane 2, peak fraction from Superdex 200 gel filtration of previous lane in 8 M urea; lane 3, peak fraction from the final RP-HPLC step; lane 4, purified PfSUB1 propeptide (PfSUB1PD). Lane M contains molecular mass markers, the sizes of which are indicated. (C) Far-ultraviolet CD spectrum of purified refolded PfSUB2PD. Deconvolution of the spectrum obtained calculated secondary structure values of 24% α-helix, 23% β-sheet, 21% reverse turn, and 32% random coil.

By analogy with processing of PfSUB1 [33] and other subtilases, conversion of PfSUB2_P_ to the 75-kDa PfSUB2_I_ form likely represents autocatalytic cleavage of the propeptide. From the mass of this species we calculated that propeptide cleavage occurs between residues Tyr680 and Lys720 of the PfSUB2 sequence. We therefore expressed in *Escherichia coli* a series of propeptide constructs of varying size, in each case extending from Asn22 (the predicted N-terminus after signal peptide removal) and terminating at different residues within the above range. Constructs were fused to a short N-terminal peptide containing a His6 tag and an S-tag (Figure 4A), an approach previously used successfully to express and characterise the PfSUB1 propeptide [33]. The protein comprising Asn22-Leu687 (called PfSUB2PD) was expressed at the highest levels, and so further work focused exclusively on this. Preliminary purification trials showed PfSUB2PD to be susceptible to degradation, so to optimise yields, much of the purification was routinely performed in the presence of 8 M urea (Figure 4B). Purified PfSUB2PD was refolded into aqueous buffer and its identity confirmed by Western blot and mass spectrometry (not shown).

Circular dichroism (CD) analysis revealed that PfSUB2PD possesses substantial secondary structure (Figure 4C). Subtilisin propeptides broadly divide into two groups according to their folding capacity; whereas some are unfolded when expressed on their own (e.g., [35]), others—including the PfSUB1 propeptide—adopt significant secondary structure in isolation [33,34,36]. The CD data indicate that the propeptide of PfSUB2 falls into this second class. Subtilase propeptides vary considerably in size and exhibit little sequence homology across evolution, but structural determination of folded propeptides from bacteria [37–39] and mammals [36] have shown that they share a core structure with a simple αβ fold. The observed αβ content of PfSUB2PD is consistent with such a structure.

**Figure 5:**
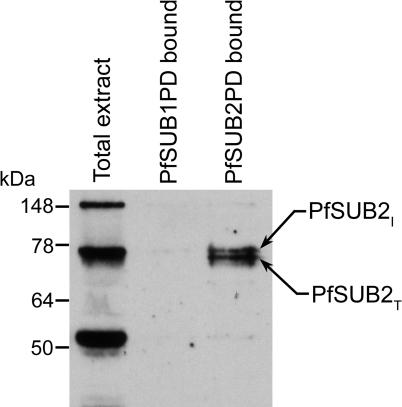
PfSUB2PD Binds to Mature Parasite PfSUB2 Sodium deoxycholate extracts of PfSUB2HA clone 2D were incubated with either purified PfSUB1PD or PfSUB2PD, then the propeptides were recovered with S-protein agarose and subjected to SDS PAGE and Western blot using anti-HA mAb 3F10. The PfSUB2_I_ and PfSUB2_T_ species bound by PfSUB2PD are indicated. Molecular mass markers are in kDa.

To seek direct evidence that PfSUB2PD was capable of binding to its cognate protease, purified PfSUB2PD was incubated with detergent extracts of PfSUB2HA clone 2D then recovered using S-protein agarose beads. Purified PfSUB1 propeptide (PfSUB1PD) was used as a control in these experiments. Figure 5 shows that of the four PfSUB2-derived species present in the detergent extracts, only PfSUB2_I_ and PfSUB2_T_—those forms predicted to lack the propeptide—were significantly bound by PfSUB2PD. Only traces of all four proteins were bound by PfSUB1PD. Reprobing these Western blots with high-titre polyclonal antibodies specific for AMA1, MSP1, or RAP2, all abundant schizont proteins, revealed no non-specific binding of any of these proteins to PfSUB2PD (not shown). These results demonstrate that PfSUB2PD can form a specific molecular complex with the mature forms of PfSUB2.

### The PfSUB2 Propeptide Is a Potent and Selective Inhibitor of MESH Activity

**Figure 6:**
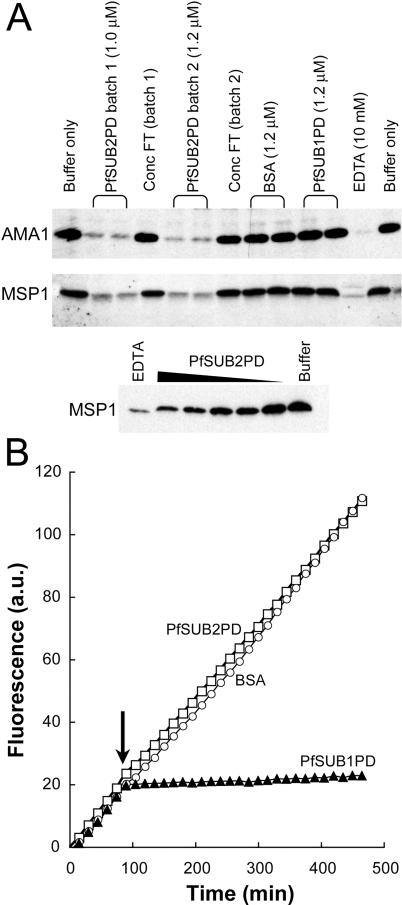
PfSUB2PD Inhibits MESH in a Selective, Dose-Dependent Manner (A) Shedding of AMA1 and MSP1 from isolated merozoites was assayed in the presence of the indicated additives. Two different batches (1 and 2) of purified PfSUB2PD were tested, in each case alongside a similar dilution of the membrane flow-through (Conc FT) from the final step of concentration by ultrafiltration. Control additives used were bovine serum albumin (BSA), purified PfSUB1PD, and EDTA. No effect on processing was seen if PfSUB2PD was added to buffer control reactions at the end of the incubation period (extreme right-hand lane). The lower panel shows a titration of inhibition of MSP1 shedding in the presence of 2-fold serially decreasing concentrations of PfSUB2PD (from 1.2 μM to 0.075 μM). (B) Effect of propeptides on PfSUB1 activity. Progress curves showing cleavage of substrate pepF1-6R by recombinant PfSUB1 before and following addition of 600 nM purified PfSUB2PD or PfSUB1PD, or BSA. Note that this concentration is in at least 300-fold molar excess over the amount of recombinant PfSUB1 present in the assay. The time point of addition of test proteins is arrowed.

Purified PfSUB2PD was then tested for its effect on MESH activity in isolated intact merozoites, taking advantage of an assay previously used extensively to characterise the inhibitor sensitivity of the sheddase. Figure 6A shows that PfSUB2PD potently inhibited shedding of both AMA1 and MSP1 in a dose-dependent manner, with a calculated IC50 (inhibitory concentration 50%) of approximately 300 nM, whilst PfSUB1PD had no detectable effect. Identical levels of inhibition were obtained with slightly less-pure preparations of PfSUB2PD, similar to those in lane 2 in Figure 4B, that had been purified in the absence of urea and had not been subjected to reversed phase–high performance liquid chromatography (RP-HPLC) (not shown).

To assess the selectivity of the inhibition mediated by PfSUB2PD, we examined its effect on the activity of recombinant PfSUB1 (Figure 6B). PfSUB2PD had no effect at the highest concentrations tested (1.2 μM), whereas PfSUB1PD rapidly abolished protease activity as reported previously [33]. To further assess whether PfSUB2PD had any capacity to act as a general serine protease inhibitor, we examined its activity against a range of other serine proteases using standard colorimetric or fluorescence-based assays. At concentrations equivalent to between 12- and 300-fold molar excess over protease, PfSUB2PD had no effect on the activity of chymotrypsin, trypsin, and elastase, though it exhibited just-detectable inhibitory activity against subtilisin Carlsberg and measurable activity against subtilisin BPN′ with a calculated K_i_ value of 1.2 μM (data not shown). Similar inhibition of heterologous subtilases by subtilase propeptides has been noted previously (e.g., [32,34]). Taken together with the binding data above, these results provide compelling evidence that the inhibition of AMA1 and MSP1 shedding mediated by recombinant PfSUB2PD is a direct result of it binding to and inhibiting the activity of mature PfSUB2 on the merozoite surface.

## Discussion

Proteolytic shedding of surface proteins during invasion by apicomplexan parasites is thought to be a strategy by which adhesion–receptor complexes are removed as the parasite enters its host cell. Shedding of MSP1 by cleavage just upstream of its C-terminal EGF-like region occurs in all species of *Plasmodium* examined, and new interest in the protease responsible was sparked by the revelation that the same protease also mediates release of another essential surface protein, AMA1. Here we have identified this protease as PfSUB2, an integral membrane merozoite subtilase.

PfSUB2 is a very minor parasite component, and in an earlier study [23], we were insufficiently confident in our antibody localisation data to assign it to a specific sub-cellular location. By epitope tagging the endogenous *pfsub2* gene and using a range of mAbs to study its expression profile compared to other well-defined markers, we have now definitively localised PfSUB2 to micronemes. This differs from the conclusions of Barale et al. (1999) [22] who used polyclonal antibodies to obtain evidence suggesting that PfSUB2 localizes to a different set of organelles called dense granules. However, cross-reactivity of polyclonal antibodies combined with the small dimensions of the malaria merozoite can render localization of sub-cellular components particularly challenging, and merozoite proteins have been previously mis-localised (e.g., [40]). A possible alternative explanation for the discrepancy between these results is that epitope tagging of PfSUB2 resulted in its mistargeting. This phenomenon has previously been observed in Apicomplexa, but seems most unlikely in this case; given the evidence that PfSUB2 is an essential gene product, a defect in sorting would likely have phenotypic consequences. Our transgenic lines and clones displayed normal growth and morphology. Importantly, moreover, our conclusions are lent credence by the finding that PfSUB2 is secreted onto the parasite surface prior to or at invasion, an activity typical of microneme proteins. Dense granule proteins, in contrast, are discharged predominantly following invasion [41].

Collectively, our findings support the following model. PfSUB2 undergoes maturation by post-translational proteolytic processing that is initiated by propeptide cleavage and results in the formation of a membrane-bound protease. By analogy with PfSUB1 maturation [25], both PfSUB2_I_ and PfSUB2_T_ are likely to be enzymatically active, but it is probably the PfSUB2_T_ form that accumulates in micronemes where it is stored until schizont rupture. At merozoite release PfSUB2 is secreted onto the parasite surface where it engages with an actin-based motor. This could occur through its cytoplasmic domain or through ectoplasmic interactions with other micronemal proteins that are themselves connected to the motor. PfSUB2 is then shuttled towards the posterior pole of the merozoite, in the process encountering and cleaving the MSP1 complex and AMA1 from the surface of the parasite. Our present results show that this capping process can occur efficiently in the absence of invasion, satisfactorily explaining the limited shedding of MSP1 and AMA1 seen in preparations of free merozoites. However, our observations may not accurately reflect the dynamics of PfSUB2 translocation at invasion. The invasive half-life of *P. falciparum* merozoites is extremely short, and shedding of MSP1 is known to go to completion during the brief (~30 s) time course of erythrocyte invasion [7]. We therefore propose that—similar to TgMIC2/M2AP—capping of PfSUB2 on the *invading* merozoite likely occurs predominantly concomitant with invasion, the protease perhaps concentrating at the moving junction. IEM studies of merozoites “captured” in the process of invasion will be required to test this postulate. Unfortunately this is a highly ambitious goal in *P. falciparum* in which, as a result of the short invasive half-life of merozoites, no surface protein has ever been localised by this technique on the invading parasite. The issue would be best addressed in the simian malaria model *P. knowlesi,* free merozoites of which are relatively long lived. This will be a major focus of future work.

An additional prediction of our model is that PfSUB2 may not be substantially exposed at the surface of the invasive merozoite prior to the point of junction formation. In support of this, we have found that addition of purified PfSUB2PD to parasite cultures has no effect on invasion (P. Harris, unpublished data). In an analogous example, antibodies against sporozoite TRAP or TgMIC2 have no effect on invasion, because these adhesins are not displayed at high levels on the parasite surface until the point of invasion, and only those molecules inhabiting the moving junction are functional during invasion [42] (V. Carruthers, personal communication). Nonetheless, we expect that it should be possible to target PfSUB2 with appropriately selective low-molecular weight, membrane-permeable compounds.

Capping of PfSUB2 to the merozoite posterior could be prevented by latrunculin A but not by cytochalasin D, a puzzling finding in view of the latter's known potent effects on invasion by the malaria merozoite and the fact that both compounds prevent invasion at the concentrations used here. Both drugs induce rapid disruption of the actin cytoskeleton in mammalian cells, but through distinct mechanisms; whereas cytochalasin D binds to filamentous actin, inhibiting polymerization at both barbed and pointed ends [43], latrunculin binds to and sequesters actin monomers, preventing their de novo polymerization [44,45]. One consequence of this is that cytochalasin D does not induce net depolymerization of actin, whereas latrunculin does [46,47], and there are now numerous examples of cellular actin-based processes that exhibit differential sensitivity to these drugs (e.g., [47,48]). Only a single actin gene is expressed in *P. falciparum* merozoites, and a recent study has shown that filaments formed by this actin are unusually short [49]. One explanation for the discrepancy between the effects observed with the two actin antagonists used in our experiments is that the degree of actin polymerisation required to provide the traction for invasion may be much greater that than required to translocate the bulk of secreted PfSUB2 the short distance (~1.2 μm) from the apical region of the merozoite to its posterior. This could explain why invasion is more sensitive than capping of PfSUB2 to perturbation by cytochalasin D.

The motility of apicomplexan zoites is stringently regulated, primarily by control of actin polymerization [50], and in highly motile apicomplexan zoites such as the *Toxoplasma* tachyzoite, motility ceases abruptly after invasion, implying rapid disassembly of the actinomyosin motor. The malaria merozoite is one of the smallest known eukaryotic cells, and so removal of the forces powering and maintaining retrograde flow of PfSUB2 after invasion would allow subsequent redistribution of the protease by simple diffusion in the parasite membrane. Our observation that PfSUB2 uniformly decorates the plasma membrane of the intracellular ring is consistent with this.

The estimated IC50 of approximately 300 nM for inhibition of MESH by PfSUB2PD is somewhat higher than K_i_ values measured for other subtilase propeptides against their cognate enzymes, which range from around 1–6 nM [32,33] to around 200 nM [51]. However, the combination of a relatively large molecular mass and the fact that it has to interact with membrane-bound protease may preclude ready access of PfSUB2PD to merozoite surface PfSUB2 in our assay. Moreover, PfSUB2PD may not represent the optimal propeptide construct; the exact internal sites at which processing takes place during PfSUB2 maturation are unknown, and it is well documented that even small truncations of propeptide constructs can compromise their inhibitory capacity (e.g., [32,33]). Despite this, PfSUB2PD exhibited clear selectivity, showing no effect on a number of serine proteases and only weak inhibition of a phylogenetically related bacterial subtilisin. A total of only three subtilases are encoded in the *Plasmodium* genome [25]. Previously, we have conclusively excluded a role for PfSUB1 in MESH activity, but one criticism of our present conclusions is that the inhibition of MESH mediated by PfSUB2PD could be the result of interactions with PfSUB3, a subtilase that is poorly characterised but known to be transcribed in parasite blood-stages [25]. However, we have recently successfully disrupted the *pfsub3* gene in blood-stages of *P. falciparum;* the resulting parasites are viable, and processing of both MSP1 and AMA1 occurs normally (R. O'Donnell and M. Blackman, unpublished data), demonstrating that PfSUB3 is not essential for MESH activity.

In *Toxoplasma gondii,* several integral membrane microneme adhesins that cap to the parasite posterior through interactions with the actinomyosin motor are shed in the final seconds of invasion via cleavage within their TMD by a class of intramembrane proteases called rhomboids [52,53]. A malaria merozoite rhomboid-like activity has also recently been identified [17], the role of which may be to shed merozoite adhesins that are functional homologues of these transmembrane adhesins [20]. The merozoite rhomboid(s) cannot be involved in juxtamembrane shedding or shedding of GPI-anchored proteins such as MSP1, because rhomboids can only cleave within a TMD [54]. A *Toxoplasma* rhomboid, TgROM5, was recently localised to the posterior surface of the tachyzoite [55], suggesting that temporal and spatial regulation of release of capped microneme proteins is achieved through the simple expedient of maintaining the protease at the rear of the parasite; substrates are effectively recruited to the protease by the capping process. Our present findings show that PfSUB2 has evolved to function in a converse fashion; because its substrates (MSP1 and AMA1) are uniformly distributed about the parasite circumference, the sheddase must be tracked across the parasite surface in order to efficiently engage with them. PfSUB2 probably acts in concert with the merozoite rhomboid(s) during invasion.

Do functional homologues of PfSUB2 exist in other Apicomplexa? Aside from the rhomboid studies alluded to above, little is known of the molecular details of shedding of surface and microneme proteins in other apicomplexan genera. Two subtilisin-like serine proteases, TgSUB1 and TgSUB2, have been identified in *T. gondii* tachyzoites, but neither of these likely performs a role analogous to that of PfSUB2; TgSUB1 is a nonessential GPI-anchored microneme protease, whereas TgSUB2 is located in rhoptries in which it appears to be involved in maturation of other rhoptry proteins [56]. It remains to be seen whether Apicomplexa other than *Plasmodium* use a subtilisin-like sheddase at invasion.

Erythrocyte invasion is an obvious but thus far underexploited target for drugs designed to block the malarial lifecycle. PfSUB2 may represent an excellent target for new protease inhibitor-based drugs, and to this end, recombinant expression of the protease in an enzymatically active form is now a priority.

## Materials and Methods

### Parasite culture and transfection.

*P. falciparum* clones D10, 3D7, and T9/96 were maintained and free merozoites produced as described [57]. For some experiments, medium was supplemented with cytochalasin D or latrunculin A (Sigma-Aldrich, Poole, United Kingdom) added from stock solutions in DMSO. To obtain highly synchronised preparations of newly invaded ring-stage parasites, mature schizonts isolated by centrifugation on cushions of 63% isotonic Percoll were cultured with fresh erythrocytes for 2 h to allow rupture and reinvasion before removal of residual schizonts as described previously [14]. For transfection, ring-stage parasites were electroporated with plasmid DNA using standard procedures [58]. Parasites containing integrated plasmid were selected by drug cycling in the presence of 10 nM WR99210 (Jacobus Pharmaceuticals, Princeton, New Jersey, United States), then cloned by limiting dilution.

### Transfection constructs.

The 3HA sequence was amplified from pREP(HA3)42 [59] using primers HA-1 5′-AGA CAC TGC TCG AGT ACC CTT ACG ATG TT-3′ and HA-2 5′-GGA CAC TGG TCG AC **T CA**A GCG TAA TCT GG −3′ (XhoI and SalI sites are underlined, and the incorporated translational stop codon is in bold). The product was ligated into the unique XhoI site just upstream of the PbDT 3′ UTR in pHH1-ΔSERA4, kindly provided by Dr. Brendan Crabb, the Walter and Eliza Hall Institute, Melbourne, Australia, [60] to produce construct pHH1-HA3. The 3′ region of the *pfsub2* gene, which contains an internal BglII site, was amplified from T9/96 *P. falciparum* genomic DNA using primers T996–1 (5′-GCC CCA GGT CAT CAC ATA TAT TCT ACT ATT CC-3′), and T996–2 (5′-GGA CAC TGC TCG AGT TTC ATA AAC ATA TCA TC-3′; XhoI site underlined); primers were designed to remove the stop codon from the end of the *pfsub2* coding sequence and form a continuous reading frame into the 3HA region. The *sera4* gene fragment was excised from pHH1-HA3 using BglII and XhoI and replaced with the BglII/XhoI-digested PCR fragment to create pHH1-T996HA3. Control plasmid pHH1-T996w was created in a similar manner but using a product amplified from T9/96 genomic DNA with primers T9961 (5′-GCC CCA GGT CAT CAC ATA TAT TCT ACT ATT C-3′) and T996_w_ (5′-CTC GAG TCA TTT CAT AAA CAT ATC ATC AAG TTG ATT CAT TGC-3′; XhoI site underlined); this amplifies a similar fragment of the 3′ end of the *pfsub2* gene but including the stop codon. The *sera4* gene fragment was excised from pHH1-ΔSERA4 using BglII and XhoI and replaced with the BglII/XhoI-digested *pfsub2* fragment. Nucleotide sequences of all cloned products were confirmed by sequencing on both strands.

### Southern blot.

Parasite genomic DNA was prepared using the DNeasy Tissue Kit (Qiagen, Valencia, California, United States) and digested using ScaI (Roche). Digested products were electrophoresed on a 0.7% agarose gel and transferred to Hybond N^+^ Nylon membrane (Amersham Biosciences, Buckinghamshire, United Kingdom). The blot was probed with a [^32^P]-labelled, gel-purified, 569-base pair PCR product extending from nucleotides 3,047 to 3,615 of the T9/96 *pfsub2* gene.

### Indirect immunofluorescence assay and fluorescence microscopy.

Thin films of *P. falciparum* cultures were acetone-fixed and incubated with a 1:500 dilution of the anti-HA mAb 12CA5 (mouse) or 3F10 (rat; Roche, Basel, Switzerland) for 30 min, then with a biotinylated goat anti-mouse or anti-rat IgG (Chemicon, Temecula, California, United States) diluted 1:500, followed by incubation with FITC streptavidin (Vector Laboratories, Burlingame, California, United States) diluted 1:500. For dual labelling, samples were additionally probed with mAb 4G2 (anti-PfAMA1; [10]), mAb 61.3 (anti-RhopH2; [61]), mAb H5 (anti-RAP2; I. Ling, unpublished data), or mAb 1E1 (anti-MSP1_19_; [62]), in each case conjugated directly to Alexa Fluor 594 (Molecular Probes, Invitrogen, Carlsbad, California, United States), or mAb X509 (anti-MSP1; [63]) followed by incubation with Alexa Fluor 594–conjugated anti-human IgG (Molecular Probes) diluted 1:500. Slides were stained with DAPI (4,6-diamidino-2-phenylindole) and mounted in Citifluor (Citifluor, Canterbury, United Kingdom). Images were collected using AxioVision 3.1 software on an Axioplan 2 Imaging system (Zeiss, Oberkochen, Germany) using a Plan-APOCHROMAT 100×/1.4 oil immersion objective, and annotated using Adobe PhotoShop.

### IEM.

Samples of late schizonts were fixed in 0.075% (v/v) double-distilled glutaraldehyde and 4% (w/v) paraformaldehyde made up in culture medium (RPMI, pH 7.2), for 20 min on ice, then washed four times in ice-cold RPMI and dehydrated through a progressively low-temperature ethanol series, infiltrated with LR White resin (EMSCOPE, London, United Kingdom) and polymerized by ultraviolet light at room temperature for 48 h. For PfSUB2HA detection, sections were immunostained with the anti-HA mAb 3F10 (diluted 1:50 or 1:100) followed by biotinylated goat anti-rat antibody (diluted 1:100) then streptavidin conjugated to 10 nm gold particles (British Biocell International, Cardiff, United Kingdom) diluted 1:100. Detection of PfAMA1 was carried out as described in a previous study [64] using a rabbit polyclonal antiserum (#680) raised against recombinant PfAMA1 (kindly donated by C. Kocken and A. Thomas, Biomedical Primate Research Centre, Rijswijk, the Netherlands). For control purposes, parallel samples were treated with irrelevant primary antibodies. Sections were stained for 4 min with 2% (w/v) aqueous uranyl acetate. Images were viewed and captured digitally with a Hitachi 7600 electron microscope.

### Expression and purification of the PfSUB2 propeptide.

A wholly synthetic gene called *pfsub2*_synth_, encoding the T9/96 PfSUB2 but lacking the intron present in the authentic *pfsub2* gene and with codon usage optimised for expression in *Trichoplusia ni* insect cells, was synthesised as described previously [65], using a total of 168 40-mer oligonucleotides. Sequence encoding Asn22-Leu687 was amplified from *pfsub2*_synth_ using forward primer 5\′-GGT ATT GAG GGT CGC AAC AAC AAC AAC AAC TAC TAC TTG-3' plus reverse primer 5\′-AGA GGA GAG TTA GAG CCC TAC AGG AAG CTG TAT TTG TTG-3' (PRO2) and cloned into the ligation-independent vector pET-30Xa/LIC (Novagen, Madison, Wisconsin, United States). The encoded propeptide construct was expressed in *E. coli* BL21-Gold (DE3) (Stratagene, La Jolla, California, United States). Cells were disrupted by sonication in 20 mM Tris-HCl, 300 mM NaCl, 20 mM imidazole (pH 8.2) supplemented with protease inhibitors, and the PfSUB2PD bound to Ni-NTA agarose (Qiagen) then eluted in the same buffer containing 250 mM imidazole. Usually, solid urea was then added to 8 M and the protein chromatographed on a Superdex 200 prep grade 26/60 column equilibrated in 20 mM Tris-HCl, 150 mM NaCl, 8 M urea, 1 mM DTT (pH 8.2). Polishing was performed on a Vydac 4.6 × 150 mm C_4_ RP-HPLC column eluted at 1 ml min^−1^ with a 45%−63% (v/v) gradient of acetonitrile in 0.1% (v/v) TFA over 20 min, monitoring elution at 215 nm. Eluted protein was taken up into gel filtration buffer and refolded by rapid 400-fold dilution into 20 mM Tris-HCl, 150 mM NaCl (pH 8.2). In some cases the gel filtration step was performed in the absence of urea, and the RP-HPLC step omitted to obtain lower yields of less-pure protein that had not been exposed to denaturing conditions. Protein was concentrated by ultrafiltration and the membrane flow-through retained as a control buffer for subsequent experiments. The identity of purified PfSUB2PD was confirmed by Western blot using an anti-polyhistidine antibody (mAb HIS-1; Sigma) and mass spectrometric peptide fingerprinting of tryptic digests [15] Its concentration was calculated from absorbance measurements at 280 nm, based on the tyrosine and tryptophan content that predicts a molar extinction coefficient of 53,305 M^−1^ cm^−1^ [66]. Purification of PfSUB1 propeptide was as described previously [33].

### CD.

CD spectra of PfSUB2PD were recorded from 250 to 190 nm at 20 °C in a fused silica cuvette of 1-mm path length using a Jasco J-715 spectropolarimeter. Protein concentrations were approximately 0.1 mg ml^−1^ in 20 mM Tris-HCl, 150 mM NaCl (pH 8.2). Spectra presented are averages of multiple scans recorded using a scan speed of 200 nm min^−1^ and a response time of 0.25 s. Buffer blanks were subtracted from all spectra. Spectra were subjected to multicomponent secondary structure analysis using the SELCON3 algorithm [67].

### Pull-down experiments and Western blot.

Percoll-enriched schizonts of PfSUB2HA clone 2D were extracted into 10 volumes of 20 mM Tris-HCl, 150 mM NaCl (pH 7.6) containing 1.5 mM sodium deoxycholate. Clarified extract (200 μl, corresponding to approximately 2 × 10^8^ schizonts) was mixed with up to 0.5-μg purified PfSUB2PD or control proteins, then further supplemented with 100-μl S-protein agarose beads (Novagen) to bind the propeptide plus any associated proteins. Beads were washed in the above buffer then bound proteins analysed by SDS PAGE and Western blot as described previously [33], using mAb 3F10 to probe the blots.

### Processing assays.

The effect of PfSUB2PD on shedding of AMA1 and MSP1 from isolated merozoites was assayed as described [16,57]. Briefly, 3D7 merozoites purified from cultures containing 10 mM EGTA were washed, suspended in 50 mM Tris-HCl, 5 mM CaCl_2_ (pH 7.6) and divided into equal aliquots of approximately 4 × 10^8^ merozoites, on ice. Aliquots were supplemented with purified PfSUB2PD, purified PfSUB1 propeptide, BSA, 10 mM EDTA or control buffers and transferred to 37 °C for 2 h. Merozoites were pelleted, and shed AMA1 or MSP1 detected in merozoite supernatants by Western blot using mAb 4G2 or mAb X509 respectively.

### Protease assays.

Production of recombinant PfSUB1 and an assay of activity based on cleavage of the fluorogenic substrate pepF1-6R has been described previously [68]. Cleavage was monitored at room temperature with a Cary Eclipse fluorescence spectrophotometer (Varian, Palo Alto, California, United States) equipped with a microplate reader accessory, using excitation and emission wavelengths of 552 nm and 580 nm respectively. Starting concentrations of substrate and PfSUB1 in these assays were 0.2 μM (well below the K_M_ for this substrate) and approximately 2 nM respectively. For other protease assays, enzymes and substrates were obtained from Sigma. Subtilisins BPN′ and Carlsberg were assayed using *N*-succinyl-L-Ala-L-Ala-L-Pro-L-Phe-*p*-nitroanilide as described previously [33]. The K_M_ value for hydrolysis of this substrate by subtilisin BPN′ under these conditions was 242 ± 8 μM. K_i_ determinations were performed as described [33]. Assays of bovine trypsin, α-chymotrypsin and porcine pancreatic elastase were performed using published procedures with substrates Nα-benzoyl-DL-Arg-7-amido-4-methylcoumarin [69], *N*-succinyl-L-Ala-L-Ala-L-Pro-L-Phe-*p*-nitroanilide [70], and *N*-succinyl-Ala-Ala-Ala-*p*-nitroanilide [71] respectively.

## Supporting Information

### Accession Numbers

The GenBank (http://www.ncbi.nlm.nih.gov/Genbank/) accession numbers for the genes discussed in this paper are *pfsub2* (AJ132422) and *pfsub2*_synth_ (AY998616).

## Abbreviations

AMA1: apical membrane antigen-1
CD: circular dichroism
GPI: glycosylphosphatidyl inositol
HA: haemagglutinin
IEM: immuno-electron microscopy
IFA: immunofluorescence
mAb: monoclonal antibody
MESH: merozoite surface sheddase
MSP1: merozoite surface protein-1
PV: parasitophorous vacuole
RP-HPLC: reversed phase–high performance liquid chromatography
TMD: transmembrane domain
